# Human pediculosis, a global public health problem

**DOI:** 10.1186/s40249-022-00986-w

**Published:** 2022-05-26

**Authors:** Yi-Tian Fu, Chaoqun Yao, Yuan-Ping Deng, Hany M. Elsheikha, Renfu Shao, Xing-Quan Zhu, Guo-Hua Liu

**Affiliations:** 1 Research Center for Parasites and Vectors, College of Veterinary Medicine, Hunan Agricultural University, Changsha, Hunan China; 2 Department of Biomedical Sciences and One Health Center for Zoonoses and Tropical Veterinary Medicine, Ross University School of Veterinary Medicine, Basseterre, Saint Kitts and Nevis; 3 Faculty of Medicine and Health Sciences, School of Veterinary Medicine and Science, University of Nottingham, Loughborough, LE12 5RD UK; 4 Centre for Bioinnovation, School of Science, Technology and Engineering, University of the Sunshine Coast, Sippy Downs, QLD 4556 Australia; 5 College of Veterinary Medicine, Shanxi Agricultural University, Taigu, 030801 Shanxi China; 6 Key Laboratory of Veterinary Public Health of Higher Education of Yunnan Province, College of Veterinary Medicine, Yunnan Agricultural University, Kunming, Yunnan 650201 People’s Republic of China

**Keywords:** Human lice, Pediculosis, Public health, Phylogenetics, Omics technology

## Abstract

**Background:**

Human pediculosis is caused by hematophagous lice, which are transmitted between individuals via direct and/or indirect contact. Despite the public health importance of louse infestation, information concerning the global burden of pediculosis and the epidemiological landscape of louse-borne diseases is limited. The aim of this review was to summarize the biology, epidemiology, diagnosis, and control of lice infestation in humans. We also discussed the latest advances in molecular taxonomy and molecular genetics of lice.

**Methods:**

We searched five electronic bibliographic databases (PubMed, ScienceDirect, CNKI, VIP Chinese Journal Database, and Wanfang Data) and followed a standard approach for conducting scoping reviews to identify studies on various aspects of human lice. Relevant information reported in the identified studies were collated, categorized, and summarized.

**Results:**

A total of 282 studies were eligible for the final review. Human pediculosis remains a public health issue affecting millions of people worldwide. Emerging evidence suggests that head lice and body lice should be considered conspecific, with different genotypes and ecotypes. Phylogenetic analysis based on mitochondrial (mt) *cyt*b gene sequences identified six distinct clades of lice worldwide. In addition to the direct effect on human health, lice can serve as vectors of disease-causing pathogens. The use of insecticides plays a crucial role in the treatment and prevention of louse infestation. Genome sequencing has advanced our knowledge of the genetic structure and evolutionary biology of human lice.

**Conclusions:**

Human pediculosis is a public health problem affecting millions of people worldwide, particularly in developing countries. More progress can be made if emphasis is placed on the use of emerging omics technologies to elucidate the mechanisms that underpin the physiological, ecological, and evolutionary aspects of lice.

**Graphic Abstract:**

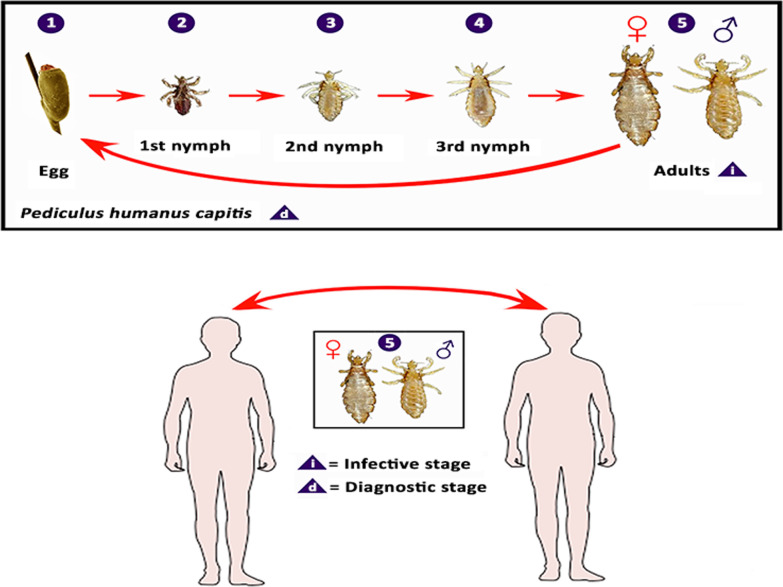

**Supplementary Information:**

The online version contains supplementary material available at 10.1186/s40249-022-00986-w.

## Background

Human pediculosis is caused by infestation of skin by blood-sucking lice [1]. Pediculosis has been known for over 10,000 years, with the oldest human louse egg found on hair from an archaeological site in north-eastern Brazil [2]. Human pediculosis remains a worldwide public health problem with an estimated 19% global prevalence of head lice among school children [3] and 2% prevalence of pubic lice in adult populations [4].

Human blood-sucking lice (Insecta: Phthiraptera: Anoplura) comprise two families Pediculidae and Pthiridae, with the corresponding genera *Pediculus* and *Pthirus*, respectively. *Pediculus humanus capitis* (*P. h. capitis*) represents the head louse; *P. h. humanus* (also known as *P. h. corporis*) represents the body louse or clothes louse; and *Pthirus pubis* refers to the pubic louse or crab louse [5]. While head lice spend their entire life on the host, body lice live mainly on the folds of the host's clothing and bedding. Head lice do not favor particular socioeconomic classes, but body lice are often observed on homeless and are associated with poverty, overcrowding and poor hygiene [6]. Crab lice prefer thick coarse hair, such as pubic hair, but can also infest other body locations [7]. Transmission of head and body lice occurs via close contact, such as head-to-head or exchanging hats, sharing pillowcases, and clothes [8]. Pubic lice are transmitted from person to person via sexual contact [9].

We performed a review of the literature to summarize data on the morphology and biology of human lice, and provide updates on the epidemiology, diagnosis, and treatment of lice infestation as well as identify areas where additional data can be useful to inform more effective control strategies.

## Methods

### Literature search strategy

**Fig. 1:**
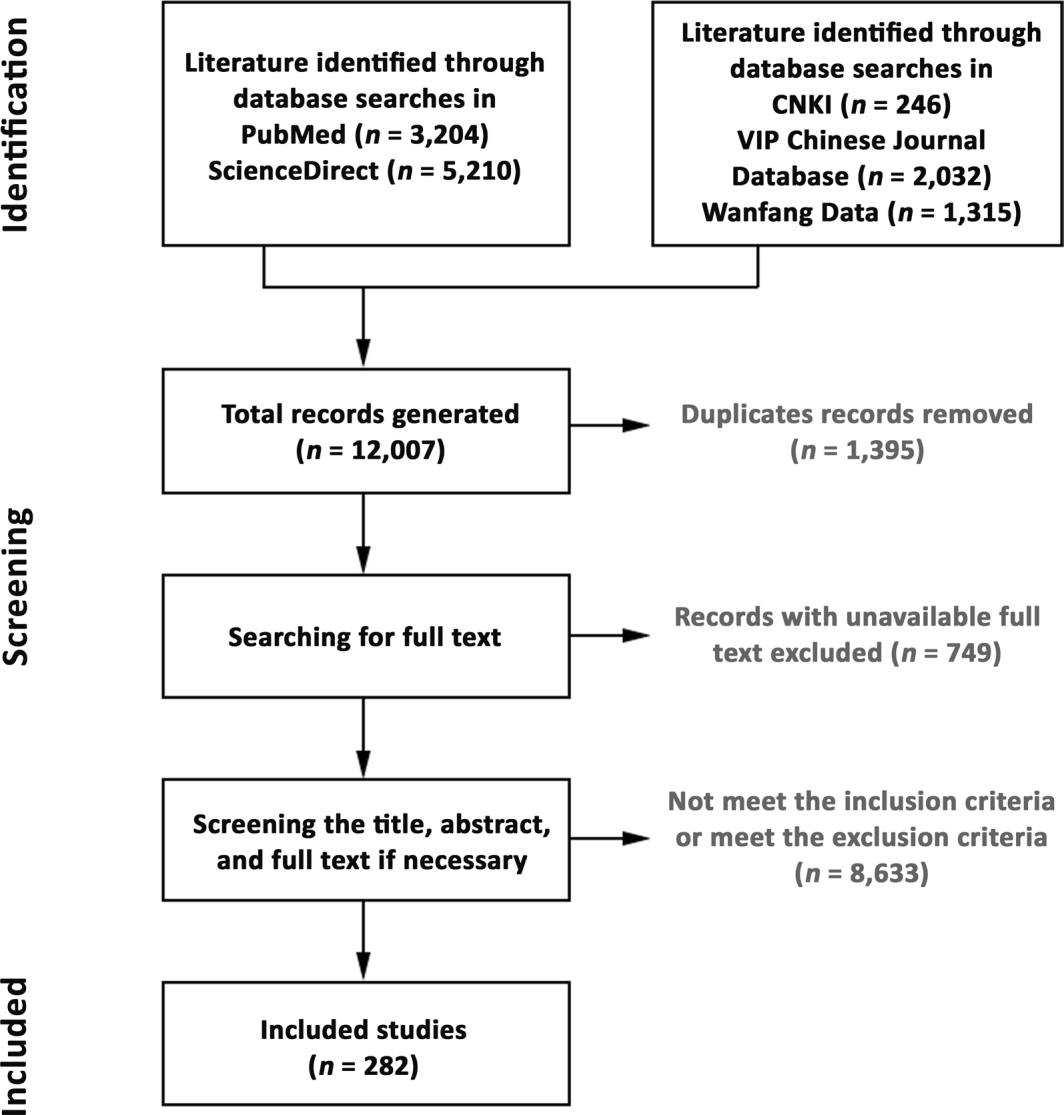
Flow diagram of the scoping review process

Literature search for English and Chinese articles published on human lice up to October 1, 2021 was performed in five electronic bibliographic databases, including PubMed, ScienceDirect, CNKI (https://www.cnki.net/), VIP Chinese Journal Database (http://qikan.cqvip.com/), and Wanfang Data (https://g.wanfangdata.com.cn/index.html). For PubMed and ScienceDirect databases, the following keywords were used: “louse”, “*Pediculus humanus*”, “head louse”, “body louse”, “pubic louse”, and “pediculosis”. In the three Chinese databases, an advanced search was carried out using Chinese translation of the keywords “human lice”, “head louse”, “body louse”, “pubic louse”, and “pediculosis”. There was no restriction on the publication year. We used Endnote (X9.2 version; Clarivate, Philadelphia, USA) to organize the articles. A flow diagram of the study selection process is shown in Fig. 1.

### Study eligibility criteria and data extraction

The study inclusion criteria included articles on human lice, have sufficient details, and with accessible full texts. Exclusion criteria included duplicate studies, studies with inaccessible full text. Screening of literature search results against the eligibility criteria was performed by two independent authors (Y-TF, Y-PD), and any disagreement was mediated by a third author (G-HL). Failure to reach a consensus was resolved by senior authors (HME, X-QZ). Two authors independently extracted and recorded data from each eligible study (Y-TF, Y-PD). The extracted data included article title, first author, publication year, infestation rate, diagnostic method, treatment, country, and host characteristics (age and gender).

## Results

### Morphological attributes of human lice

**Fig. 2:**
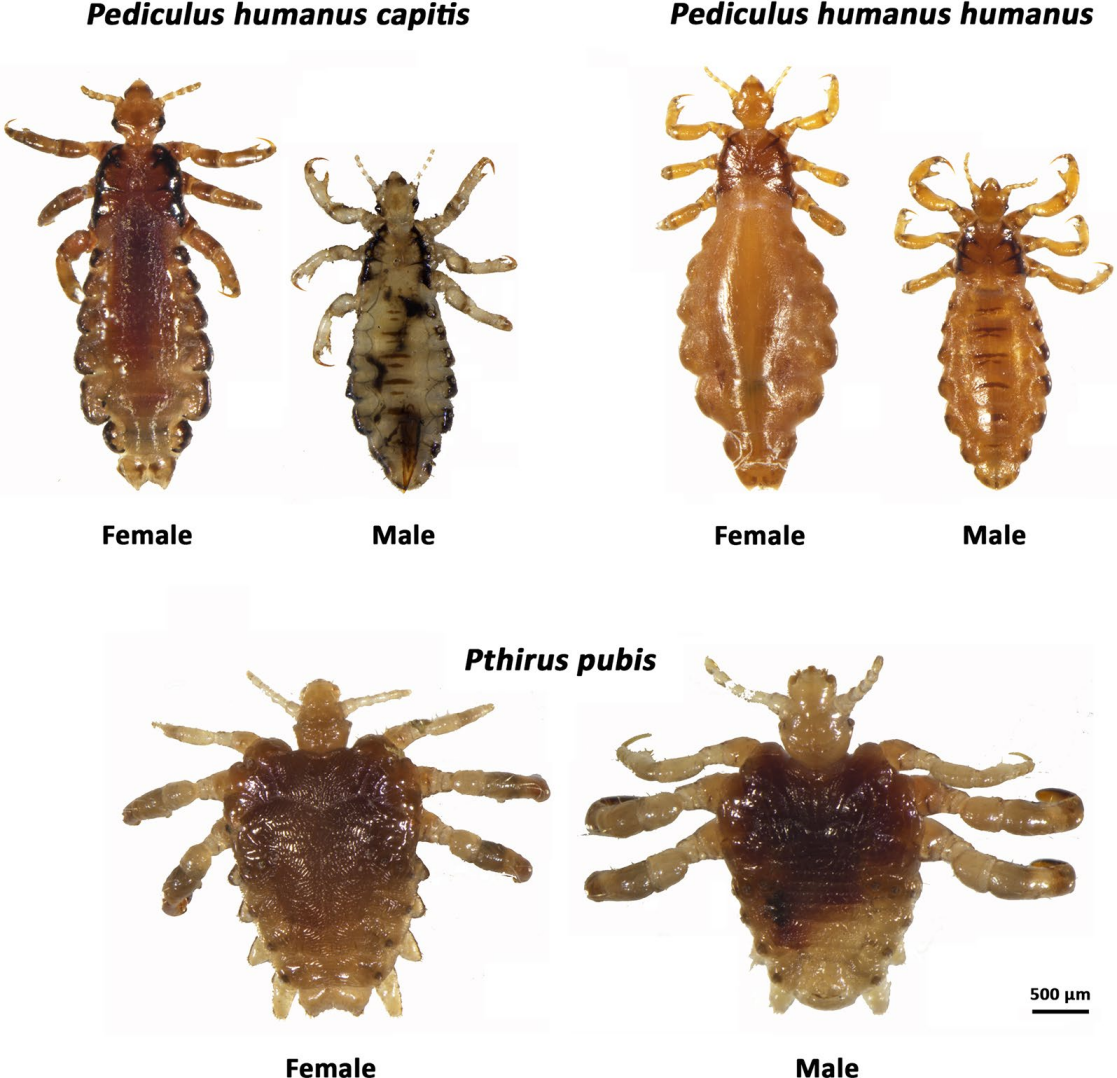
Human lice

Human lice are blood-sucking insects and use delicate stylets to probe and pierce the host’s skin to reach the blood vessels and inject saliva with anticoagulant properties to increase the flow of blood during blood feeding [10]. Identification and differentiation of lice have been traditionally based on morphological features. Pubic louse can be easily distinguished from head louse and body louse based on morphology. Head louse and body louse are almost morphologically indistinguishable, although the former is slightly smaller than the latter (Fig. 2) [11, 12]. A recent study showed subtle morphological differences at the antenna level between head louse and body louse [13]. Head lice are ovoid, 2–3 mm long, and grayish white in colour. Body lice are 2.3–3.6 mm in length. Although some phenotypic features are different between head louse and body louse, many of these morphological differences can be attributed to the distortion of the flexible exoskeleton during sample dehydration and mounting [14]. Human lice are wingless and dorsoventrally flattened insects with three pairs of clawed legs adapted for grasping the hair. Their narrow sucking mouthparts are hidden inside the head which also bears a pair of short antennae [11]. Females are slightly larger than males. Louse eggs, known as ‘nits’, are transparent, flask-shaped, and 0.5 mm in size [15]. Unlike the ovoid-shape of the head and body louse, the crab louse is almost as wide as long, enabling it to grasp widely spaced pubic hairs [11]. Pubic lice are 0.8–1.2 mm long and sturdy. They have three pairs of legs, the first pair is short and delicate, whereas the other two pairs terminate in prominent crab-like claws that firmly hold pubic or other body hairs [11, 16].

### Taxonomy and phylogenetics of human lice

The taxonomic status of pubic louse is well established. However, taxonomy of head lice and body lice has been debated for more than two centuries [17–20], and both species are generally considered conspecific [21–25]. The nuclear and mitochondrial (mt) DNA sequences have been used to identify lineages and analyze genetic variations in *P. humanus* (Additional file 1: Table S1). Mitochondrial genes are suitable markers for genotyping of *P. humanus* due to their unique characteristics, including maternal inheritance, high copy numbers, fast evolutionary rate, simple genetic structure, and lack of recombination [25, 26].

**Fig. 3:**
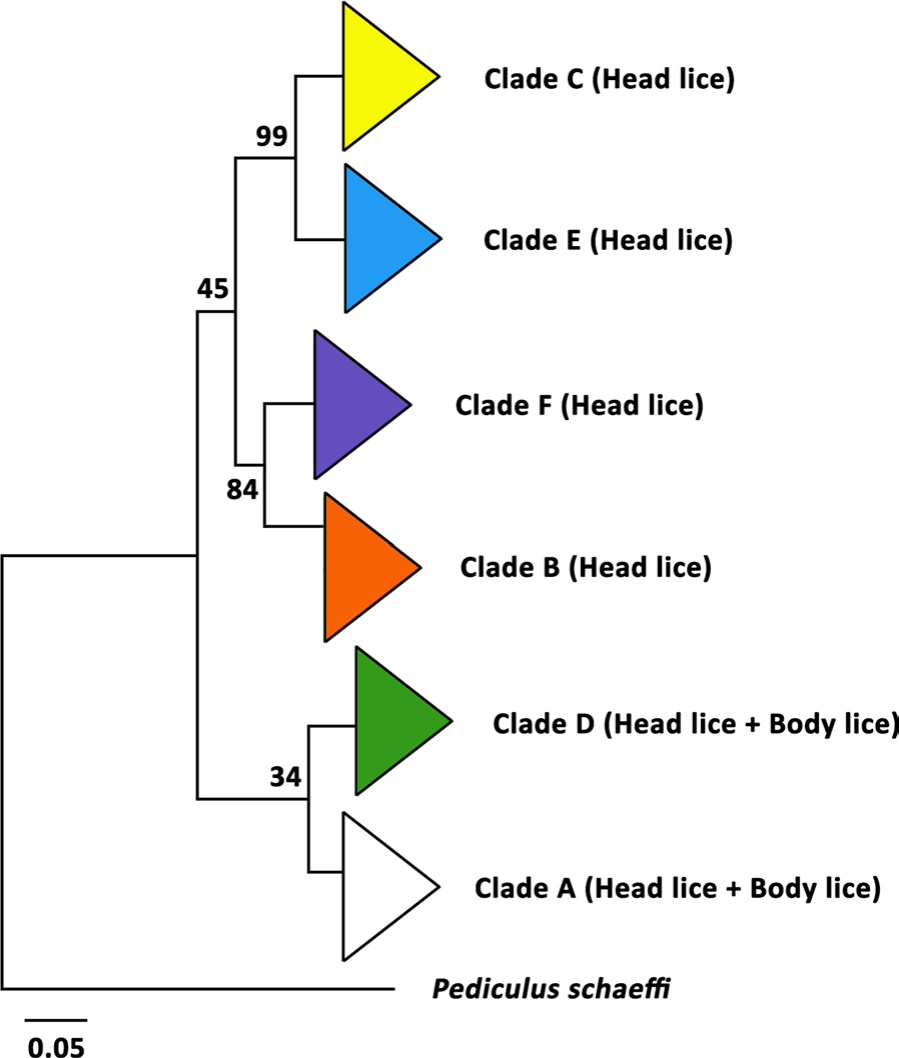
Phylogenetic analysis of the mitochondrial *cyt*b sequences of *Pediculus humanus* from different geographical regions and countries. The sequences of *cyt*b were aligned using MAFFT 7.245, and neighbor-joining (NJ) phylogenetic tree was constructed using MEGA 6, with the GTR + I + G substitution model selected by jModelTest 2.1.7. Bootstrap frequency (Bf) was calculated using 100 bootstrap replicates. *Pediculus schaeffi* was used as an outgroup. Scale bar denotes nucleotide substitutions per site

Phylogenetic analysis of the mt *cyt*b gene grouped lice into six distinct clades, A to F (Additional file 1: Table S2; Fig. 3). Interestingly, head lice appeared in all clades whereas body lice belonged only to clades A and D [25, 27], supporting the notion that body lice have evolved from head lice [28], and suggesting that lice in the other clades never transferred from head to clothing during the prehistoric or historic past. Clade A included lice found worldwide (Table 1) [1]. Clade B included lice found in America, Australia, Israel, Algeria, South Africa, Saudi Arabia, and Western Europe. Clade C included lice found in Africa (Ethiopia, Senegal, and Republic of Congo) and Asia (Nepal, Pakistan, and Thailand). Clade D included lice found in Ethiopia, Republic of Congo, and Zimbabwe. Clade E comprised head lice from Guinea and West Africa (Mali and Senegal).

**Table 1:** Worldwide distribution of human louse *Pediculus humanus* clades

Clade	Ecotype	Continent	Country/region
A	*Pediculus humanus capitis*, *Pediculus humanus humanus*	All over the world	All over the world
B	*Pediculus humanus capitis*	Africa	Algeria, South Africa
		Asia	Israel, Saudi Arabia
		Australia	Australia
		Europe	Austria, Belgium, Bulgaria, Croatia, Czech Republic, Denmark, France, Finland, Germany, Greece, Hungary, Iceland, Italy, Macedonia, Montenegro, Netherlands, Portugal, Romania, Serbia, Slovakia, Slovenia, Spanish, Sweden, Switzerland, United Kingdom
		Latin America	Belize, Costa Rica, Guatemala, Honduras, Mexico, Nicaragua, Panama, Peru, Salvador
		North America	Canada, The United States
C	*Pediculus humanus capitis*	Africa	Ethiopia, Senegal, the Republic of Congo
		Asia	Nepal, Pakistan, Thailand
		Europe	France
D	*Pediculus humanus capitis*, *Pediculus humanus humanus*	Africa	Ethiopia, the Democratic Republic of Congo, the Republic of Congo, Zimbabwe
E	*Pediculus humanus capitis*	Africa	Guinea, Mali, Senegal
F	*Pediculus humanus capitis*	Latin America	Argentina, Mexico, French Guiana

The most newly discovered clade F is a sister group of clade B (Fig. 3) [25] and included head lice mainly from Native American individuals in French Guiana. Clade F also included a few sequences of lice from Argentina and Mexico [25]. The recovery of ancient head louse nits from six shrunken human heads further supports the hypothesis of a native South American origin of clade F [29]. *P. mjobergi*, a louse of South American monkeys of the Cebidae family, also belongs to this clade. It was speculated that this louse was carried by people who migrated to the New World and then adapted to monkeys, and thus, may represent evolutionary lineage of *P.* *humanus* [25, 28]. Taken together, these data support the notion that head lice and body lice have shared tracks of human migration [27, 30], and that lice have co-evolved with humans [31]. However, caution should be exercised with the interpretation of louse phylogeny because extraction of DNA was never done on voucher specimens taxonomically identified in some previous studies (see Additional file 1: Table S2).

### Human louse biology

**Fig. 4:**
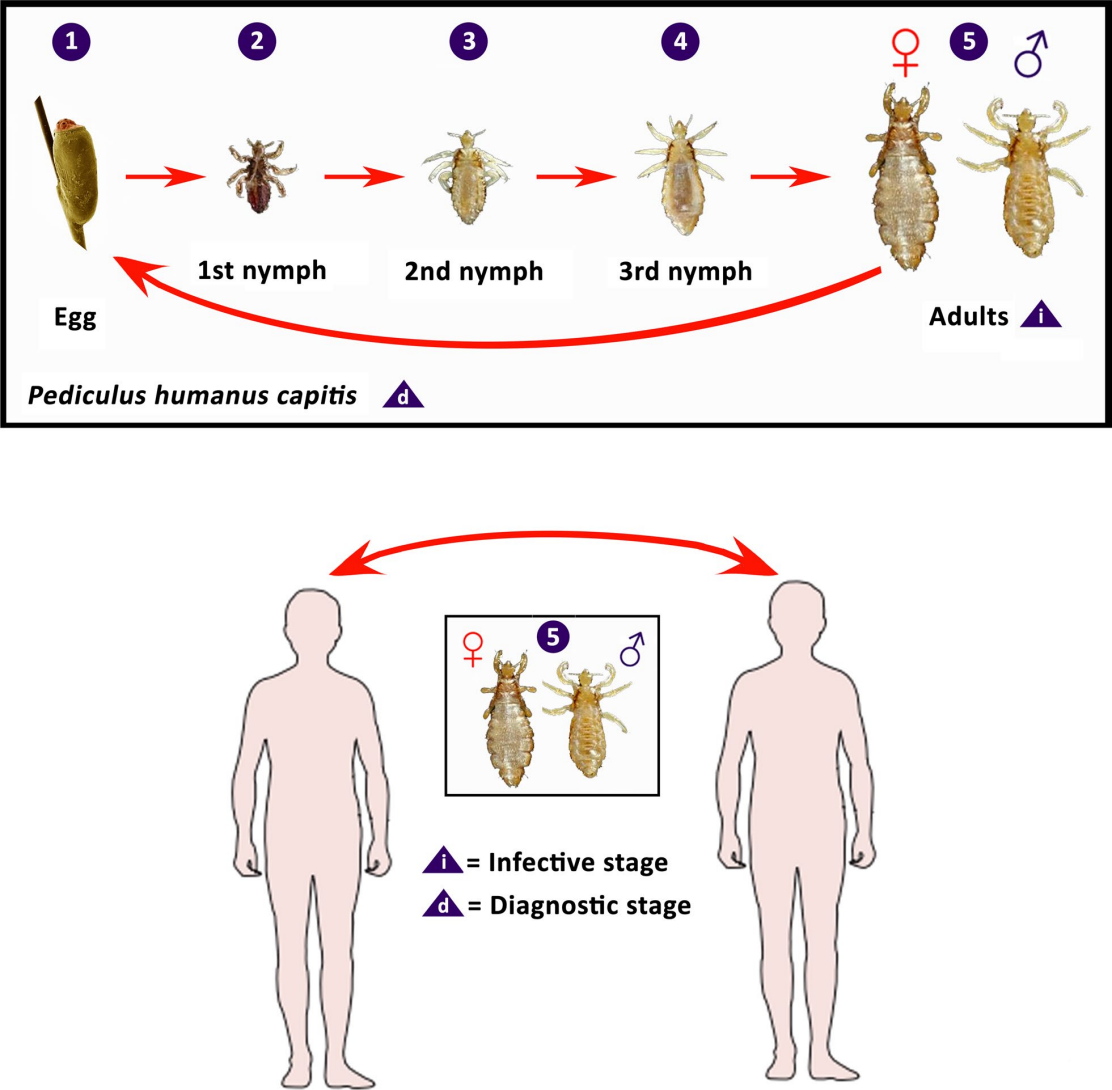
Life cycle of human head louse, *Pediculus humanus capitis*. Adapted from Centers for Disease Control and Prevention (CDC) DPDx website (https://www.cdc.gov/parasites/lice/head/biology.html)

Louse life cycle includes egg, three nymphal stages, and adult (Fig. 4). Human lice require regular blood meals to survive and complete their development. A female louse lays eggs which are glued to the scalp hair (head lice), cloth folds (body lice), or pubic hair (pubic lice) of the host [32]. Bacot [17] observed that the daily average number of eggs produced by an adult head louse was four, which was consistent with Nuttall’s finding [33], but Lang [34] noted a higher fecundity of head lice (average 6.6 ± 3.9 viable eggs/day). Later studies showed that an adult female head louse laid 50–150 eggs during its lifespan of about 16 days [11]. A body louse lays approximately 270–300 eggs over 18 days [11]. However, a simplified protocol for in vitro rearing of body lice showed that a female louse lays approximately 10.8 eggs during 30 days, with 94% hatchability [35]. A pubic louse lays 3–10 eggs per day and fixes ≤ 30 eggs to hair in a lifetime [4]. Lice are sensitive to temperature and humidity. For example, body lice survive best in 79–90% humidity and at 29–32 ℃. They die rapidly in an environment with humidity < 40% or temperature > 50 ℃ [36]. Louse eggs are usually laid in locations with optimal temperature and humidity. For example, eggs of head lice are found on the scalp, particularly around and behind the ears or near the neckline [37], while body louse eggs are found on the seam of clothing close to the skin, and eggs of pubic lice can be found on the hairs of the chest, abdomen, legs, and buttocks [11]. Climatic conditions can influence the position of eggs. For example, head lice generally lay eggs within 1 mm of the scalp to maximize eggs’ development and hatching at ambient temperature. However, in the warm humid climate, eggs can be found eight or more inches from long hairs that lay across the scalp and are close to the skin [38].

After about a week for head lice or 8–10 days for body lice [10], the first nymph hatches from eggs, leaving the shells attached to hair or clothing seams. The nymphs take a blood meal facilitated by chemical compounds with anticoagulant and vasodilator properties in their saliva [39]. Human lice take frequent blood meals, approximately every 3–4 h [37]. They mature into adults after three molts, which takes about 9–12 days for head lice and over 2 weeks for body lice and pubic lice [11]. The lifespan of a louse is about one month on the host [40]. In the environment, head lice and body lice can only survive for one or two days, respectively. Eggs removed from a host can only survive for up to 10 days in conditions of high humidity and at a temperature above 28 ℃.

### Epidemiology

#### Head lice

Head louse infestation has historically been and is likely to remain a worldwide problem because head lice can infest people of all ages, and various social and economic status [11]. Surveys of head louse prevalence have been summarized in previous reviews [40, 41]. A survey of public health records from 1910 to 1930 in Glasgow, United Kingdom, reported prevalence as high as 50% in some communities [42]. The prevalence of head louse has significantly declined in developed countries due to the use of medications [43]. In developing countries, and places where insecticides are not readily available, the prevalence has probably remained unchanged [40]. Since the 1970s, the prevalence of lice has increased in many countries, and hundreds of millions of people have been infested globally, ranging from zero to 78.6% in different countries and areas [3, 41]. A school survey of head lice in Benghazi, Libya, revealed an alarmingly high prevalence of 78.6% [44]. In the USA, the affected population is estimated to be 6–12 million annually with school children being the main victims [37].

Head lice seem to be common in children. Children between three and 12 years old often have the highest prevalence as they are more likely to interact with each other, particularly at school [11]. The prevalence of head lice in girls is higher than in boys, probably because girls are more likely to come into close contact [45]. Having a long and brown hair has also been reported as risk factors for head lice infestation in children [46]. More transfer of head louse between children and women who look after them can be expected because they are more likely to come into direct contact.

Race may be another factor affecting louse prevalence. A survey conducted in two elementary schools in the United States showed that Caucasian children had the highest prevalence, follow by Hispanic children and African American children, whereas Asian children had the lowest prevalence [47]. A study of head lice among primary school children in Kenya showed that black children had lower prevalence than non-black children [48]. Other factors can also influence louse prevalence, such as season, educational level, family size and pesticide resistance [39, 47]. Repeated infestations can occur; a record of 15–19 infestations annually had been detected in Brazilian patients [37].

#### Body lice

Body lice are predominantly prevalent in the homeless people, refugees and people living in crowded and/or unsanitary conditions [11, 32]. They are transmitted among humans via close body-to-body contact, and their prevalence often reflects the socioeconomic status of the infested population [16]. Body louse outbreaks occur mostly during conflicts or natural disasters with a prevalence as high as 90–100% reported during the civil wars in Burundi, Rwanda, and Zaire in the 1990s [32]. In some developing countries, infestation rate 11–22% was detected in sheltered homeless people [32]. Co-infestation by head lice and body lice occurred in 4.3% of homeless people in San Francisco, USA and in 59% of street children in Kathmandu and Pokhara, Nepal [32].

#### Pubic lice

Although less information is available about the distribution of crab lice, there is an indication that this species is widely distributed [19]. Pubic lice are mainly transmitted via sexual contact and are therefore more prevalent in adults than in children. The latter may occasionally become infested via contact with bedding and towels. Approximately 2% of adults are infested worldwide [49, 50]. Infested people tend to be 15–40 years old with a mean age of 30.3 years. Those over 35 years old only account for 18.8% of all patients [51]. Prevalence in women peaks at 15–19 years old and in men at 20 years old and older [51]. Men are more likely to be infested than women [51]. Interestingly, incidence of pubic louse infestation starts to decline as more people opt to shave their pubic hairs [52], which could be an effective method of control and prevention among highly risk populations. Co-infestation of the scalp by both pubic louse and head louse is possible, although rare [53].

### Clinical manifestations

The most common clinical symptoms are pruritus, papular urticaria, excoriations, and cervical/occipital lymphadenopathy [15]. For head lice, considerable variations exist in the development of pruritus, with 36% of cases report itching [54] and 14.2% of the cases are symptomless [55]. Most of pediculosis symptoms in school children are mild to moderate; although rare, heavy and chronic infestations can lead to anaemia [56]. In addition to physical signs, head louse infestation causes psychological stress in children and parents [56] and an outbreak in school may lead to frustration among parents [57].

Body louse infestation causes generalized pruritus and lesions are generally found on the neck, shoulders, upper back, flanks, and waist—sites of close contact between clothing and skin [15]. Common lesions include excoriation and eczematous patches; papular urticaria has also be reported [15]. Prurigo nodules, lichenification, and hyperpigmentation are found in chronic infestation. Scratching predisposes to impetigo, ecthyma and cellulitis [15].

Pubic lice cause intense itching in the pubic region. They are typically located at pubis, groin, buttocks, intergluteal fold and perianal region [58]. They may also appear on the thighs, face (beard and/or eyelash), abdomen, chest, axillae, and scalp, mainly in hairy males and/or in long-lasting infestations [59]. Itching may produce erythema, small skin ulcerations and secondary bacterial infections; scratching also induces erythematous sores [50, 51]. Infestations of the eyelashes may result in pthiriasis palpebrarum [60] and other ocular symptoms, including a follicular conjunctivitis and mild punctate epitheliopathy [61]. It is worth mentioning that pthiriasis of eyelashes may suggest a sexually transmitted infestation, usually occurs in children and may suggest child abuse [59, 62]. It is also more likely related to close contact with infested individuals or contaminated clothing, towels, and bedding. Pubic lice occurring in the scalp is called “pthiriasis capitis” and is commonly associated with eczematous and pruritic lesions on the nape [59]. Maculae ceruleae can appear on the lower abdomen and thighs – with the colour of the maculae being associated with deep dermal hemosiderin deposition from the louse bites [11].

### Lice as vectors of disease-causing agents

#### Head lice

Head lice belonging to clade C were found positive for the spirochaete *Borrelia recurrentis*, the causative agent of louse-borne relapsing fever (LBRF) [63]. *B. theileri*, the agent of relapsing fever-like illness, has been detected in head lice in Congo [24]. *B. quintana*, the etiological pathogen of trench fever, was found in head lice from Senegal and Ethiopia [64–66]. A laboratory study showed that head lice are capable of transmitting *Rickettsia prowazekii* [67, 68]. Head lice might be a competent natural vector of rickettsiae [69]. *Acinetobacter baumannii* has been detected in head lice in Ethiopia, France and Thailand [70–72]. *Acinetobacter* spp. have been frequently found in head lice collected from school children [73, 74], however, their clinical relevance remains to be confirmed [24]. DNA of *Yersinia pestis* has been detected in head lice belonging to clades A and D in plague-endemic areas [23, 75]. DNAs of other pathogenic bacteria have also been detected in head lice (Table 2). More studies are needed to explore clade-specific differences in the vectorial capacity of head lice and the mechanisms underlying these variations.

**Table 2:** Pathogens associated with *Pediculus humanus* and *Pthirus pubis* lice

	Head lice (*Pediculus humanus capitis*)	Body lice (*Pediculus humanus humanus*)	Pubic lice (*Pthirus pubis*)
DNA detection	*Borrelia recurrentis* *Bartonella quintana* *Borrelia* spp. *Rickettsia prowazekii* *Acinetobacter* spp. *Yersinia pestis* *Serratia marcescens* *Coxiella burnetii* *Rickettsia aeschlimannii* *Anaplasma* sp. *Ehrlichia* spp. *Moraxellaceae* *Staphylococcus* spp. *Streptobacillus moniliformis* *Haemophilus influenzae* *Bordetella pertussis*	*Rickettsia prowazekii* *Borrelia recurrentis* *Bartonella quintana* *Yersinia pestis* *Acinetobacter* spp. *Serratia marcescens* *Coxiella burnetii* *Rickettsia* spp. *Anaplasma phagocytophilum* *Ehrlichia muris*	*Acinetobacter johnsonii* *Acinetobacter* spp. *Rickettsia prowazekii,* *Bartonella quintana*
	*Acinetobacter baumannii*		
	*Bartonella quintana*		
Experimental model	*Bartonella quintana* *Rickettsia prowazekii*	*Yersinia pestis* *Rickettsia rickettsia* *Rickettsia conorii* *Rickettsia typhi* *Rickettsia akari* *Acinetobacter lwoffii* *Acinetobacter baumannii*	No reported cases
Disease transmission	No reported cases	Trench fever Louse-borne relapsing fever Epidemic typhus	No reported cases

#### Body lice

Body lice can transmit *R. prowazekii*, *B. recurrentis* and *B. quintana*, the causative agents of epidemic typhus, relapsing fever and trench fever, respectively [76]. *B. recurrentis* was associated with the “Yellow Plague” pandemic in the sixth century [77] and continues to be a threat to the vulnerable populations living in poor hygienic conditions.

During World War I, trench fever was widely spread among soldiers deployed to regions with poor hygienic practices, and more than one million people were affected [78]. *B. quintana* has been detected in 13.1–28.2% of body lice collected from residents in San Francisco, Bogotá, Algiers, Tizi Ouzou, Boumerdès and Gaziantep [79–82]. In recent years, the prevalence of *B. quintana* in body lice in homeless in Marseille has decreased from 21.0 to 1.2% [83, 84].

In 1909, Charles Nicolle reported that body lice could carry *R. prowazekii*, one of the epidemic typhus-causing bacteria. Outbreaks of typhus fever have been reported during Russian Revolution and World War II [76] and in recent years in Burundi and Russia [32]. Body lice may play a role in transmitting plague. Under a laboratory condition, three *Y. pestis* strains were transmitted between rabbits by body lice [85]. *Y. pestis* has been detected in body lice during plague outbreaks [75, 85]. Furthermore, paleomicrobiological evidence indicated that body lice may have contributed to plague pandemics [86].

Body lice may also serve as a potential vector of other pathogens (Table 2). *Acinetobacter* spp. DNA has been detected in homeless people in Marseille, especially DNA of *A. baumannii* [74], which is an opportunistic antibiotic-resistant bacterium, often associated with nosocomial infections [87]. These studies suggest that body lice may carry a broad spectrum of pathogens.

#### Pubic lice

*A. johnsonii* was first detected in pubic lice in Boudouaou, Algeria [73]. *A. johnsonii* has attracted public attention due to its resistance to carbapenem and co-aggregation during wastewater treatment [88–90]. Others *Acinetobacter* spp. were identified in pubic lice collected from Marseille and Bobigny [91]. *P. pubis* can be successfully infected by *B. quintana* and *R. prowazekii* under laboratory conditions. *B. quintana* DNA was detected in pubic lice in Bobigny, suggesting that pubic lice could serve as a vector for *B. quintana* [91]. Pubic louse associated pathogens are listed in Table 2.

### Diagnosis of louse infestation

Louse infestation is usually diagnosed based on visual detection of the adult and nymphal stages [92]. The presence of louse eggs and eggs’ shell does not necessarily indicate active infestation [93]. Louse eggs should be examined microscopically for viable embryos [93]. Checking for head lice should be performed by direct visual inspection with the aid of a fine-toothed comb. A comb should be inserted through the hair down to the scalp, starting from hair roots and combing through from shaft to tip. This method can be as twice fast and four times more effective than visual inspection alone [16]. Wet combing is preferred as moisture slows down louse movement. Combing should focus on sites such as the left and right temples, behind ears and neck [94]. Eggs located within 6 cm of the scalp should draw attention, because head lice usually lay eggs on the hair shaft close to the scalp, which take 6‒10 days to hatch and hair grows by 0.4 mm daily [15, 16].

Infestation by body lice is diagnosed by finding lice and eggs on clothes, focusing on the parts that come into contact with body regions of high temperature such as inner belt of the underwear, bands of trousers or skirts, side seams and underarm seams [11]. Atopic dermatitis, allergic dermatitis, drug reaction or viral exanthem rashes may manifest during early infestation [11]. Heavy infestation may lead to iron-deficiency anemia and eosinophilia [15].

Pubic louse infestation is diagnosed by visual detection of lice or nits. Because of their small size, they are usually detected with the help of a magnifying glass [93] or dermoscopy [95, 96]. It is important to note that pthiriasis capitis caused by *P. pubis* on the scalp can be misdiagnosed as pediculosis capitis caused by head louse. Microscopical examination of lice collected from the scalp is thus essential for correct etiological diagnosis [59, 97].

Misdiagnosis may occur when the lice are damaged or preserved. Vacuuming hair and scalp seem less effective than visual inspection [98]. MALDI-TOF MS has been proposed as a quick, and accurate tool for identification of lice stored in alcohol for different durations of time [99]. Analysis of 18S rRNA and mt genes has been an effective method to accurately diagnose lice if specimens are badly damaged [73, 91]. Morphological attributes can differentiate between *Pediculus* lice and *P. pubis* lice. However, body lice and head lice are morphologically indistinguishable, but can be differentiated by multiplex real-time PCR [100]. Although molecular techniques can accurately delineate the differences between body lice and head lice, they are labor-intensive and not suitable for routine applications in medical practice.

### Treatment and management of louse infestation

Treatment of head lice involves insecticides and mechanical removal [15]. Topical therapies are summarized in Table 3. Over-the-counter treatments include permethrin, pyrethrin/piperonyl butoxide (PB), malathion 0.5% lotion, spinosad, ivermectin 5% lotion and oral use, lindane, benzyl alcohol 5% lotion, and dimethicone. Essential oils have also been used for controlling head lice [101]. Abametapir offers a safe and effective treatment option for head lice infestation [102]. Lindane is no longer recommended due to safety concerns [103]. Oral ivermectin can be used for treatment of head lice, particularly in individuals with recurrent infestations [104].

**Table 3:** Topical insecticides and/or medications used in the management of head louse infestation

Insecticide	Drug class	Mechanism of action	Application	Efficacy %	Side effects	References
Permethrin	Synthetic pyrethroid	Inhibits sodium ion influx	Infants (> 2 months) and adults	20–30% eggs remained	Irritation and allergy	[16, 132, 134]
Piperonyl butoxide	Benzodioxoles	Inhibits the metabolism of pyrethrin and increases its persistence and effectiveness against lice	Children (> 2 years) and adults	78–83% (combined with permethrin)	Dermatological symptoms	[16, 132, 134]
Dimethicone	Polydimethylsiloxane	Physical action	Children (> 1 year) and adults	> 80% (for pyrethroid-resistant)	Dermatological symptoms	[135–137]
Xeglyze	Abametapir	Inhibits metalloproteinase	Infants (> 6 months) and adults	Prevented 100% of eggs from hatching	Scalp erythema, rash and burning sensation	[102]
Lotilaner	Isoxazoline	Inhibitsγ-aminobutyric acid-gated chloride channels	School children and older	100% death rate for adult lice with 10 and 100 μmol/L after 3 h exposure	Dermatological symptoms	[138]
Malathion (0.5%)	Organophosphate	Inhibits cholinesterase and acute toxicity	Children (> 6 years)	82–100%	Scalp dryness, irritation, dandruff	[16, 103, 134, 139–141]
Spinosad	Aminoglycosides	Alters function of acetylcholine and GABA-gated ion channels	Children (> 4 years)	68–87%	Breathing problems	[6, 142]
Ivermectin 5%	Macrocyclic lactone	Activates glutamate-gated chloride channels in nerve and muscle cells	Children (> 6 months) and adults	74%	Eye irritation and skin-burning sensation	[104, 143, 144]
Oral ivermectin	Macrocyclic lactone	Activates glutamate-gated chloride channels in nerve and muscle cells	Children aged > 5 years and/or weighing > 15 kg and adults	77–95%	Impetigo, nausea or vomiting, gastroenteritis, and convulsion	[141, 145, 146]
Benzyl alcohol 5%	Aromatic alcohol	Closing respiratory spiracles and suffocation	Infants (> 6 months) and adults	75–76%	Breathing problems	[16, 134, 143]

Body louse infestation can be controlled via adopting good personal hygiene and frequent changes of clean, washed clothing. Additional measures include washing clothing, towels, and bed linens with hot water (e.g., 50 °C for 30 min or shorter times at higher temperatures) [105, 106]. Antibiotics are needed in case of louse-borne infections [107]. Pubic-louse infestation requires treatment of the patients and their partners [108, 109]. Topical 1% permethrin or 0.3% pyrethrin is the first-line medication [109] in addition to the Food and Drug Administration (FDA)—approved 0.5% ivermectin lotion [15]. Petrolatum jelly is useful for treating eyelash infestation [110]. Oral ivermectin is a second-line medication, yet to be approved by FDA [4, 15].

Treatment failure can be attributed to louse resistance to insecticides [111–120] or incorrect use of product [121], including too short exposure time, insufficient dosage or applying solution onto dripping-wet hairs. Unless an ovicide product is used, treatment must be repeated after seven days to kill newly hatched nymphs. In addition to the use of insecticides, other control measures are necessary for effective control of louse infestation. For example, machine laundering with hot water at 50 ℃ or above can help in eradicating head lice [122]. All washable cloths and beddings used by an infested person should be treated by tumble drying. Separation of personal items at school is necessary and children should be encouraged to keep their personal items, such as hats and scarves, separated from one another [11]. Parents and school nurses should regularly check children for lice. For pubic louse infestation, both sexual partners should be treated [7]. Physical contact between infested person(s) and their partner(s) should be avoided until full cure has been achieved. Public lice cases in children should be reported due to the possibility of sexual exploitation.

### Insight gleaned from molecular biology and new omics technologies

The mt genome sequences of head, body and pubic lice are available. These data are crucial for reconstructing human-lice phylogeny. The human lice mt genomes contain 37 genes including two rRNA genes, 22 tRNA genes, and 13 protein-coding genes [123]. These are located on 20, 20 and 15 minichromosomes, respectively [123, 124], rather than locating on a single megachromosome as in vertebrate animals. Each minichromosome, often 3–4 kb in length, carries one to five genes in addition to a non-coding region (NCR) [125]. Recombination plays an important role in the mt genome fragmentation across louse clades [126]. Head louse and body louse in clade A are 97.8% identical in the coding regions of the mt genomes. Their gene content and arrangement have remained unchanged since they are separated from one another approximately 107,000 years ago despite substantial variations in NCR’s sequence and size, contrarily, distribution of *P. pubis* mt tRNA genes is different from that of *P. humanus* [125].

Nuclear (18S rRNA, EF-1α) or mt (*cox*1, *cyt*b, *nad*4) gene sequences have been used to determine the genotypes, ecotypes, and haplotypes of lice. Molecular techniques such as quantitative real-time polymerase chain reaction (qPCR) and DNA sequencing can be used to identify human louse species and/or to trace their evolutionary history [22, 24, 28, 127]. Genomic analyses have also been used to detect pathogens transmitted by lice [1, 91].

Body lice have the smallest genome of all insects reported to date, with 108 Mb in female and 109 Mb in male lice [128]. The genome is AT-rich (72%) and contains 10,773 protein-coding genes and 57 microRNA genes. Body lice have significantly fewer protein-coding genes associated with environmental sensing compared with other insects’ genomes [128] and fewer gene-coding proteins necessary for host location and selection, such as odorant and gustatory receptors, odorant-binding proteins, and chemosensory proteins [129]. Their genomes also contain the fewest genes encoding detoxifying enzymes among insects [129]. Intriguingly, body lice have a single gene for insulin-like peptide, which may reflect its restricted diet [129]. Transcriptomic analysis showed that only one gene is differentially expressed between head and body lice [130]. A total of 3,598 alternative splicing events are specific to head or body lice [131]. Exon skipping alternative splicing events are overrepresented, whereas intron retention events are underrepresented in head and body lice [131].

## Discussion

Human pediculosis is a public health concern affecting millions of people worldwide, particularly in developing countries. Infestations by head and pubic lice have significant psychological and medical impacts not only on the affected individuals but also on their families and friends [108, 132]. Emerging data on louse morphology [1, 28] and molecular genetics [125, 128, 130] indicate that head lice and body lice are indistinguishable and should be considered conspecific, with different genotypes and ecotypes. More comparative genomic and phylogenetic analyses are needed to assess the level of genetic differences between head lice and body lice, and to examine whether they emerged via recent divergent evolutionary events.

In recent years, there has been an increasing recognition of the important role of lice in the transmission of various bacterial pathogens. Whether head louse and pubic louse serve as a natural vector of *B. quintana* and *Acinetobacter* spp. remains to be confirmed. The advances in omics technologies have revolutionized many scientific disciplines and can be a useful complement to the currently used methods to improve the understanding of the mechanisms and factors that mediate the louse's vectorial capacity and their interaction with the various pathogens they transmit. Likewise, more investigations into comparative genomics of human lice can advance our understanding of the implications of and mechanisms that underpin the evolution of mt chromosome into multiple minichromosomes.

The use of insecticides plays a crucial part in the treatment and prevention of louse infestation. However, repeated insecticide applications can contribute to the emergence of resistance. Indeed, the efficacy of many insecticides has been reduced by the emergence of insecticide-resistant lice, which to some extent underpins the increasing incidence of head louse infestations in many geographic regions [111]. Dichloro-diphenyl-trichloroethane resistance was first reported in body lice in Korea [112] and subsequently pyrethroid resistance was reported in head louse in other countries [113, 114]. Louse resistance to insecticides is mediated by different mechanisms, such as knockdown resistance (kdr) to permethrin and resistance to malathion via enhanced carboxylesterase activity [111, 116]. Head louse resistance to pyrethroid is attributed to kdr and is associated with three-point mutations and amino acid substitutions, M815I, T917I, and L920F, of the voltage-sensitive sodium channel α subunit [114, 117]. New mutations, K794E, F815I, and N818D, involving other amino acid substitutions have also been reported [120]. Ivermectin resistance involves three non-synonymous mutations (A251V, H272R, and S46P) in GluCl [118].

Challenges associated with the control of resistant lice highlight the need for more in depth understanding of the mechanisms of insecticide resistance, which may also lead to identification of new targets for the development and/or optimization of more effective compounds [115, 133]. Recent advances in omics (genomics, transcriptomics, proteomics, and metabolomics) technologies can enable detailed characterization of any biological system at an unprecedented level and can therefore facilitate the understanding of the mechanism (s) that drive resistance of lice to insecticides. For example, proteomic analysis of ivermectin-resistant lice identified 22 differentially regulated proteins; of those, 13 proteins were upregulated and nine were downregulated. The neuronal protein complexin was the most significantly downregulated protein, suggesting its potential role in regulating ivermectin resistance [119].

## Conclusions

Despite the extensive studies reported on human pediculosis in many geographical regions, the global public health and economic burden of pediculosis remains largely unknown. Public awareness will help in tackling barriers to implementation of effective treatment and control programs. Preventive measures should consider improvement of the assessment of risk of infestation and targeted resource allocation to treat the most vulnerable populations. Control of lice will also safeguard public health against louse-borne diseases. Utilization of genomics and other omics technologies can advance our knowledge about lice from their biology and physiology to genetic structure and evolution, and inform future epidemiological, diagnostic, and therapeutic innovations.

## Supplementary Information

**Additional file 1: Table S1.** Summary of the main genetic studies reported on human head and body lice. **Table S2.** Species of head lice and body lice included in mt *cyt*b gene phylogenetic analysis.

## Data Availability

Not applicable.

## Abbreviations

Mm: Millimeter
Mt: Mitochondrial
*Cyt*b: Cytochrome b
LBRF: Louse-borne relapsing fever
Cm: Centimeter
MALDI-TOF MS: Matrix-assisted laser desorption ionization time-of-flight mass spectrometry
rRNA: Ribosomal RNA
PCR: Polymerase chain reaction
PB: Piperonyl butoxide
Min: Minute
FDA: Food and Drug Administration
tRNA: Transfer RNA
NCR: Non-coding region
EF-1α: Elongation factor-1α
*Cox*1: Cytochrome c oxidase subunit 1
*Nad*4: NADH dehydrogenase subunit 4
qPCR: Quantitative real-time polymerase chain reaction
Kdr: Knockdown resistance
GluCl: Glutamate-gate chloride

## References

[1] Amanzougaghene N, Fenollar F, Raoult D, Mediannikov O (2020). Where are we with human lice? A review of the current state of knowledge. Front Cell Infect Microbiol.

[2] Araújo A, Ferreira LF, Guidon N, Maues Da Serra Freire N, Reinhard KJ, Dittmar K (2000). Ten thousand years of head lice infection. Parasitol Today..

[3] Hatam-Nahavandi K, Ahmadpour E, Pashazadeh F, Dezhkam A, Zarean M, Rafiei-Sefiddashti R (2020). Pediculosis capitis among school-age students worldwide as an emerging public health concern: a systematic review and meta-analysis of past five decades. Parasitol Res.

[4] Patel PU, Tan A, Levell NJ (2021). A clinical review and history of pubic lice. Clin Exp Dermatol.

[5] Light JE, Allen JM, Long LM, Carter TE, Barrow L, Suren G (2008). Geographic distributions and origins of human head lice (*Pediculus **humanus** capitis*) based on mitochondrial data. J Parasitol.

[6] Izri A, Guiguen C (2013). Pediculosis and laboratory role. Rev Francophone Lab.

[7] Heukelbach J, Feldmeier H (2004). Ectoparasites—the underestimated realm. Lancet.

[8] Rózsa L, Apari P (2012). Why infest the loved ones—inherent human behaviour indicates former mutualism with head lice. Parasitology.

[9] Chosidow O (2000). Scabies and pediculosis. Lancet.

[10] Nutanson I, Steen C, Schwartz RA (2007). Pediculosis corporis: an ancient itch. Acta Dermatovenerol Croat.

[11] Ko CJ, Elston DM (2004). Pediculosis. J Am Acad Dermatol.

[12] Jin D (1999). Taxonomy and fauna of sucking lice (Anoplura) in China.

[13] Ortega-Insaurralde I, Picollo MI, Barrozo RB (2021). Sensory features of the human louse antenna: new contributions and comparisons between ecotypes. Med Vet Entomol.

[14] Ferris GF (1935). Contributions towards a monograph of the sucking lice. Biol Sci.

[15] Coates SJ, Thomas C, Chosidow O, Engelman D, Chang AY (2020). Ectoparasites: pediculosis and tungiasis. J Am Acad Dermatol.

[16] El-Bahnasawy MM, Abdel FE, Morsy TA (2012). Human pediculosis: a critical health problem and what about nursing policy?. J Egypt Soc Parasitol.

[17] Bacot AW (1917). A contribution to the bionomics of *Pediculus **humanus* (vestimenti) and *Pediculus capitis*. Parasitology.

[18] Nuttall GHF (1919). The biology of *Pediculus*
*humanus*, supplementary notes. Parasitology.

[19] Buxton PA (1947). The louse.

[20] Busvine JR (1978). Evidence from double infestations for the specific status of human head and body lice (Anoplura). Syst Entomol.

[21] Boutellis A, Abi-Rached L, Raoult D (2014). The origin and distribution of human lice in the world. Infect Genet Evol.

[22] Ashfaq M, Prosser S, Nasir S, Masood M, Ratnasingham S, Hebert PD (2015). High diversity and rapid diversifification in the head louse, *Pediculus **humanus* (Pediculidae: Phthiraptera). Sci Rep.

[23] Drali R, Shako JC, Davoust B, Diatta G, Raoult D (2015). A new clade of African body and head lice infected by *Bartonella **quintana* and *Yersinia pestis*-Democratic Republic of the Congo. Am J Trop Med Hyg.

[24] Amanzougaghene N, Akiana J, Mongo Ndombe G, Davoust B, Nsana NS, Parra HJ (2016). Head lice of pygmies reveal the presence of relapsing fever borreliae in the Republic of Congo. PLoS Negl Trop Dis.

[25] Amanzougaghene N, Fenollar F, Davoust B, Djossou F, Ashfaq M, Bitam I (2019). Mitochondrial diversity and phylogeographic analysis of *Pediculus **humanus* reveals a new Amazonian clade "F". Infect Genet Evol.

[26] Mokhtar AS, Ling Lau Y, Wilson JJ, Abdul-Aziz NM (2020). Genetic diversity of *Pediculus **humanus** capitis* (Phthiraptera: Pediculidae) in Peninsular Malaysia and molecular detection of its potential associated pathogens. J Med Entomol.

[27] Amanzougaghene N, Fenollar F, Sangaré AK, Sissoko MS, Doumbo OK, Raoult D (2017). Detection of bacterial pathogens including potential new species in human head lice from Mali. PLoS One.

[28] Veracx A, Raoult D (2012). Biology and genetics of human head and body lice. Trends Parasitol.

[29] Amanzougaghene N, Charlier P, Fenollar F, Raoult D, Mediannikov O (2022). Putative native South Amerindian origin of head lice clade F: evidence from head lice nits infesting human shrunken heads. Sci Rep.

[30] Li W, Ortiz G, Fournier PE, Gimenez G, Reed DL, Pittendrigh B (2010). Genotyping of human lice suggests multiple emergencies of body lice from local head louse populations. PLoS Negl Trop Dis.

[31] Pedersen MW, Antunes C, De Cahsan B, Moreno-Mayar JV, Sikora M, Vinner L (2022). Ancient human genomes and environmental DNA from the cement attaching 2000-year-old head lice nits. Mol Biol Evol..

[32] Badiaga S, Brouqui P (2012). Human louse-transmitted infectious diseases. Clin Microbiol Infect.

[33] Nuttall GHF (1917). The biology of *Pedicuius*
*humanus*. Parasitology.

[34] Lang JD (1975). Biology and control of the head louse, *Pediculus **humanus** capitis* (Anoplura: Pediculidae) in a semi-arid urban area.

[35] Pietri JE, Ray R (2020). A simplified protocol for *in vitro* rearing of human body lice. Parasite.

[36] Sangaré AK, Doumbo OK, Raoult D (2016). Management and treatment of human lice. Biomed Res Int.

[37] Bohl B, Evetts J, McClain K, Rosenauer A, Stellitano E (2015). Clinical practice update: pediculosis capitis. Pediatr Nurs.

[38] Meinking TL (2003). Infestations. Curr Probl Dermatol.

[39] Weems HV, Fasulo TR. Human lice: body louse, *Pediculus humanus humanus* Linnaeus and head louse, *Pediculus humanus capitis* De Geer (Insecta: Phthiraptera (Anoplura): Pediculidae). The document of EENY-103, 104, Entomology and Nematology Department, Florida Cooperative Extension Service, Institute of Food and Agricultural Sciences, University of Florida; 2017.

[40] Burgess IF (1995). Human lice and their management. Adv Parasitol.

[41] Gratz NG. Human Lice. Their prevalence, control and resistance to insecticides. WHO/CTD/WHOPES/97.8. Geneva: WHO. 61 pp. 1997.

[42] Lindsay SW (1993). 200 years of lice in Glasgow: an index of social deprivation. Parasitol Today.

[43] Mellanby K (1943). The incidence of head lice in England. Med Officer.

[44] Bharija SC, Kanwar AJ, Singh G, Belhaj MS (1988). Pediculosis capitis in Benghazi, Libya. A school survey. Int J Dermatol.

[45] AlBashtawy M, Hasna F (2012). Pediculosis capitis among primary-school children in Mafraq Governorate, Jordan. East Mediterr Health J.

[46] Willems S, Lapeere H, Haedens N, Pasteels I, Naeyaert JM, De Maeseneer J (2005). The importance of socio-economic status and individual characteristics on the prevalence of head lice in schoolchildren. Eur J Dermatol.

[47] Estrada JS, Morris RI (2000). Pediculosis in a school population. J Sch Nurs.

[48] Chunge RN (1986). A study of head lice among primary schoolchildren in Kenya. Trans R Soc Trop Med Hyg.

[49] Anderson AL, Chaney E (2009). Pubic lice (Pthirus pubis): history, biology and treatment vs knowledge and beliefs of US college students. Int J Environ Res Public Health.

[50] Dholakia S, Buckler J, Jeans JP, Pillai A, Eagles N, Dholakia S (2014). Pubic lice: an endangered species?. Sex Transm Dis.

[51] Varela JA, Otero L, Espinosa E, Sánchez C, Junquera ML, Vázquez F (2003). *Phthirus** pubis* in a sexually transmitted diseases unit: a study of 14 years. Sex Transm Dis.

[52] Armstrong NR, Wilson JD (2006). Did the "Brazilian" kill the pubic louse?. Sex Transm Infect.

[53] Singh S, Singh N, Ray JC, Roy S, Garg SP (1990). *Phthirus** pubis* infestation of the scalp: report of three cases. Rev Infect Dis.

[54] Mumcuoglu KY, Klaus S, Kafka D, Teiler M, Miller J (1991). Clinical observations related to head lice infestation. J Am Acad Dermatol.

[55] Maunder JW (1993). An update on head lice. Health Visit.

[56] Rassami W, Soonwera M (2012). Epidemiology of pediculosis capitis among schoolchildren in the eastern area of Bangkok, Thailand. Asian Pac J Trop Biomed.

[57] Leung AK, Fong JH, Pinto-Rojas A (2005). Pediculosis capitis. J Pediatr Health Care.

[58] Burgess I, Maunder JW, Myint TT (1983). Maintenance of the crab louse, *Pthirus** pubis*, in the laboratory and behavioural studies using volunteers. Community Med.

[59] Veraldi S, Scanni G, Nazzaro G (2020). "Eczema" of the nape: a marker of pthiriasis capitis. Parasitol Int.

[60] Mumcuoğlu KY (2015). Pubic louse (*Pthirus** pubis*) infestation of the scalp in a 4-years old infant. Chonnam Med J.

[61] Rundle PA, Hughes DS (1993). *Phthirus** pubis* infestation of the eyelids. Br J Ophthalmol.

[62] Skinner CJ, Viswalingam ND, Goh BT (1995). *Phthirus** pubis* infestation of the eyelids: a marker for sexually transmitted diseases. Int J STD AIDS.

[63] Boutellis A, Mediannikov O, Bilcha KD, Ali J, Campelo D, Barker SC (2013). *Borrelia **recurrentis* in head lice, Ethipia. Emerg Infect Dis.

[64] Boutellis A, Veracx A, Angelakis E, Diatta G, Mediannikov O, Trape JF (2012). *Bartonella **quintana* in head lice from Sénégal. Vector Borne Zoonotic Dis.

[65] Cutler S, Abdissa A, Adamu H, Tolosa T, Gashaw A (2012). *Bartonella **quintana* in Ethiopian lice. Comp Immunol Microbiol Infect Dis.

[66] Diatta G, Mediannikov O, Sokhna C, Bassene H, Socolovschi C, Ratmanov P (2014). Prevalence of *Bartonella **quintana* in patients with fever and head lice from rural areas of Sine-Saloum, Senegal. Am J Trop Med Hyg.

[67] Goldberger J, Anderson JF (1912). The transmission of typhus fever, with especial reference to transmission by the head louse (*Pediculus capitis*). Public Health Rep Wash..

[68] Murray ES, Torrey SB (1975). Virulence of *Rickettsia prowazekii* for head lice. Ann NY Acad Sci.

[69] Robinson D, Leo N, Prociv P, Barker SC (2003). Potential role of head lice, *Pediculus **humanus** capitis*, as vectors of *Rickettsia prowazekii*. Parasitol Res.

[70] Bouvresse S, Socolovshi C, Berdjane Z, Durand R, Izri A, Raoult D (2011). No evidence of *Bartonella **quintana* but detection of *Acinetobacter **baumannii* in head lice from elementary schoolchildren in Paris. Comp Immunol Microbiol Infect Dis.

[71] Kempf M, Abdissa A, Diatta G, Trape JF, Angelakis E, Mediannikov O (2012). Detection of *Acinetobacter **baumannii* in human head and body lice from Ethiopia and identification of new genotypes. Int J Infect Dis.

[72] Sunantaraporn S, Sanprasert V, Pengsakul T, Phumee A, Boonserm R, Tawatsin A (2015). Molecular survey of the head louse *Pediculus **humanus** capitis* in Thailand and its potential role for transmitting *Acinetobacter* spp. Parasit Vectors.

[73] Mana N, Louni M, Parola P, Bitam I (2017). Human head lice and pubic lice reveal the presence of several *Acinetobacter* species in Algiers. Algeria Comp Immunol Microbiol Infect Dis.

[74] Boumbanda-Koyo CS, Mediannikov O, Amanzougaghene N, Oyegue-Liabagui SL, Imboumi-Limoukou RK, Raoult D (2020). Molecular identification of head lice collected in Franceville (Gabon) and their associated bacteria. Parasit Vectors.

[75] Piarroux R, Abedi AA, Shako JC, Kebela B, Karhemere S, Diatta G (2013). Plague epidemics and lice, Democratic Republic of the Congo. Emerg Infect Dis.

[76] Barbieri R, Drancourt M, Raoult D (2021). The role of louse-transmitted diseases in historical plague pandemics. Lancet Infect Dis.

[77] Warrell DA (2019). Louse-borne relapsing fever (*Borrelia **recurrentis* infection). Epidemiol Infect.

[78] Ruiz J (2018). *Bartonella **quintana*, past, present, and future of the scourge of World War I. APMIS.

[79] Bonilla DL, Cole-Porse C, Kjemtrup A, Osikowicz L, Kosoy M (2014). Risk factors for human lice and bartonellosis among the homeless, San Francisco, California, USA. Emerg Infect Dis.

[80] Faccini-Martínez ÁA, Márquez AC, Bravo-Estupiñan DM, Calixto OJ, López-Castillo CA, Botero-García CA (2017). *Bartonella **quintana* and typhus group Rickettsiae exposure among homeless persons, Bogotá. Colombia Emerg Infect Dis.

[81] Louni M, Mana N, Bitam I, Dahmani M, Parola P, Fenollar F (2018). Body lice of homeless people reveal the presence of several emerging bacterial pathogens in northern Algeria. PLoS Negl Trop Dis.

[82] Ulutasdemir N, Eroglu F, Tanrıverdi M, Dagli EI, Koltas IS (2018). The epidemic typhus and trench fever are risk for public health due to increased migration in southeast of Turkey. Acta Trop.

[83] De Liberato C, Magliano A, Romiti F, Menegon M, Mancini F, Ciervo A (2019). Report of the human body louse (*Pediculus **humanus*) from clothes sold in a market in central Italy. Parasit Vectors.

[84] Ly TDA, Amanzougaghene N, Hoang VT, Dao TL, Louni M, Mediannikov O (2020). Molecular evidence of bacteria in clothes lice collected from homeless people living in shelters in Marseille. Vector Borne Zoonotic Dis.

[85] Houhamdi L, Lepidi H, Drancourt M, Raoult D (2006). Experimental model to evaluate the human body louse as a vector of plague. J Infect Dis.

[86] Raoult D (2016). A personal view of how paleomicrobiology aids our understanding of the role of lice in plague pandemics. Microbiol Spectr.

[87] Howard A, O'Donoghue M, Feeney A, Sleator RD (2012). *Acinetobacter **baumannii*: an emerging opportunistic pathogen. Virulence.

[88] Malik A, Sakamoto M, Ono T, Kakii K (2003). Coaggregation between *Acinetobacter*
*johnsonii* S35 and *Microbacterium*
*esteraromaticum* strains isolated from sewage activated sludge. J Biosci Bioeng.

[89] Lee M, Woo SG, Ten LN (2012). Characterization of novel diesel-degrading strains *Acinetobacter **haemolyticus* MJ01 and *Acinetobacter **johnsonii* MJ4 isolated from oil-contaminated soil. World J Microbiol Biotechnol.

[90] Figueiredo S, Bonnin RA, Poirel L, Duranteau J, Nordmann P (2012). Identification of the naturally occurring genes encoding carbapenem-hydrolysing oxacillinases from *Acinetobacter **haemolyticus*, *Acinetobacter **johnsonii*, and *Acinetobacter **calcoaceticus*. Clin Microbiol Infect.

[91] Amanzougaghene N, Mediannikov O, Ly TDA, Gautret P, Davoust B, Fenollar F (2020). Molecular investigation and genetic diversity of *Pediculus* and *Pthirus* lice in France. Parasit Vectors.

[92] Roberts RJ (2002). Clinical practice. Head lice. N Engl J Med.

[93] Flinders DC, De Schweieggz P (2004). Pediculosis and scabies. Am Fam Physician.

[94] Feldmeier H (2012). Pediculosis capitis: new insights into epidemiology, diagnosis and treatment. Eur J Clin Microbiol Infect Dis.

[95] Jimenez-Cauhe J, Fernandez-Nieto D, Ortega-Quijano D, Ramos-Rodriguez D (2020). Characterization of *Phthirus** pubis* with ex vivo dermoscopy. Sex Transm Dis.

[96] Li L, Liu X, Xu L, Lu Y (2018). Dermoscopy of pediculosis pubis. JAAD Case Rep.

[97] Signore RJ, Love J, Boucree MC (1989). Scalp infestation with *Phthirus** pubis*. Arch Dermatol.

[98] Lustosa BPR, Haidamak J, Oishi CY, Souza AB, Lima BJFS, Reifur L (2020). Vaccuuming method as a successful strategy in the diagnosis of active infestation by *Pediculus **humanus** capitis*. Rev Inst Med Trop Sao Paulo.

[99] Benyahia H, Ouarti B, Diarra AZ, Boucheikhchoukh M, Meguini MN, Behidji M (2021). Identification of lice stored in alcohol using MALDI-TOF MS. J Med Entomol.

[100] Drali R, Boutellis A, Raoult D, Rolain JM, Brouqui P (2013). Distinguishing body lice from head lice by multiplex real-time PCR analysis of the Phum_PHUM540560 gene. PLoS One.

[101] Soonwera M, Wongnet O, Sittichok S (2018). Ovicidal effect of essential oils from Zingiberaceae plants and *Eucalytus** globulus* on eggs of head lice *Pediculus **humanus** capitis *De Geer. Phytomedicine.

[102] Woods AD, Porter CL, Feldman SR (2022). Abametapir for the treatment of head lice: a drug review. Ann Pharmacother.

[103] Verma P, Namdeo C (2015). Treatment of pediculosis capitis. Indian J Dermatol.

[104] Sanchezruiz WL, Nuzum DS, Kouzi SA (2018). Oral ivermectin for the treatment of head lice infestation. Am J Health Syst Pharm.

[105] Maunder JW (1977). Human lice—biology and control. J R Soc Promo Health.

[106] Maunder JW (1983). Pediculosis corporis; an updating of attitudes. Environ Health-Glob.

[107] Sangaré AK, Rolain JM, Gaudart J, Weber P, Raoult D (2016). Synergistic activity of antibiotics combined with ivermectin to kill body lice. Int J Antimicrob Agents.

[108] Workowski KA, Bachmann LH, Chan PA, Johnston CM, Muzny CA, Park I (2021). Sexually transmitted infections treatment guidelines, 2021. MMWR Recomm Rep.

[109] Salavastru CM, Chosidow O, Janier M, Tiplica GS (2017). European guideline for the management of pediculosis pubis. J Eur Acad Dermatol Venereol.

[110] Ma DL, Vano-Galvan S (2010). Infestation of the eyelashes with *Phthirus** pubis*. CMAJ.

[111] Durand R, Bouvresse S, Berdjane Z, Izri A, Chosidow O, Clark JM (2012). Insecticide resistance in head lice: clinical, parasitological and genetic aspects. Clin Microbiol Infect.

[112] Hurlbut HS, Altman RM, Nibley C (1952). DDT resistance in Korean body lice. Science.

[113] Downs AM (2004). Managing head lice in an era of increasing resistance to insecticides. Am J Clin Dermatol.

[114] Larkin K, Rodriguez CA, Jamani S, Fronza G, Roca-Acevedo G, Sanchez A (2020). First evidence of the mutations associated with pyrethroid resistance in head lice (Phthiraptera: Pediculidae) from Honduras. Parasit Vectors.

[115] Clark JM, Yoon KS, Kim JH, Lee SH, Pittendrigh BR (2015). Utilization of the human louse genome to study insecticide resistance and innate immune response. Pestic Biochem Physiol.

[116] Kwon DH, Kim JH, Kim YH, Yoon KS, Clark JM, Lee SH (2014). Identification and characterization of an esterase involved in malathion resistance in the head louse *Pediculus **humanus** capitis*. Pestic Biochem Physiol.

[117] Roca-Acevedo G, Del Solar Kupfer CP, Dressel Roa P, Toloza AC (2019). First determination of pyrethroid knockdown resistance alleles in human head lice (Phthiraptera: Pediculidae) from Chile. J Med Entomol.

[118] Amanzougaghene N, Fenollar F, Diatta G, Sokhna C, Raoult D, Mediannikov O (2018). Mutations in GluCl associated with field ivermectin-resistant head lice from Senegal. Int J Antimicrob Agents.

[119] Amanzougaghene N, Fenollar F, Nappez C, Ben-Amara A, Decloquement P, Azza S (2018). Complexin in ivermectin resistance in body lice. PLoS Genet.

[120] GhahvechiKhaligh F, Djadid ND, Farmani M, AsadiSaatlou Z, Frooziyan S, Abedi Astaneh F (2021). Molecular monitoring of knockdown resistance in head louse (Phthiraptera: Pediculidae) populations in Iran. J Med Entomol.

[121] Meister L, Ochsendorf F (2016). Head Lice. Dtsch Arztebl Int.

[122] Izri A, Chosidow O (2006). Efficacy of machine laundering to eradicate head lice: recommendations to decontaminate washable clothes, linens, and fomites. Clin Infect Dis.

[123] Shao R, Kirkness EF, Barker SC (2009). The single mitochondrial chromosome typical of animals has evolved into 18 minichromosomes in the human body louse, *Pediculus **humanus*. Genome Res.

[124] Fu YT, Dong Y, Wang W, Nie Y, Liu GH, Shao R (2020). Fragmented mitochondrial genomes evolved in opposite directions between closely related macaque louse *Pedicinus*
*obtusus* and colobus louse *Pedicinus*
*badii*. Genomics.

[125] Shao R, Zhu XQ, Barker SC, Herd K (2012). Evolution of extensively fragmented mitochondrial genomes in the lice of humans. Genome Biol Evol.

[126] Feng S, Pozzi A, Stejskal V, Opit G, Yang Q, Shao R (2022). Fragmentation in mitochondrial genomes in relation to elevated sequence divergence and extreme rearrangements. BMC Biol.

[127] Boutellis A, Bitam I, Fekir K, Mana N, Raoult D (2015). Evidence that clade A and clade B head lice live in sympatry and recombine in Algeria. Med Vet Entomol.

[128] Kirkness EF, Haas BJ, Sun W, Braig HR, Perotti MA, Clark JM (2012). Genome sequences of the human body louse and its primary endosymbiont provide insights into the permanent parasitic lifestyle. Proc Natl Acad Sci USA.

[129] Lee SH, Kang JS, Min JS, Yoon KS, Strycharz JP, Johnson R (2010). Decreased detoxification genes and genome size make the human body louse an efficient model to study xenobiotic metabolism. Insect Mol Biol.

[130] Olds BP, Coates BS, Steele LD, Sun W, Agunbiade TA, Yoon KS (2012). Comparison of the transcriptional profiles of head and body lice. Insect Mol Biol.

[131] Tovar-Corona JM, Castillo-Morales A, Chen L, Olds BP, Clark JM, Reynolds SE (2015). Alternative splice in alternative lice. Mol Biol Evol.

[132] Frankowski BL, Weiner LB (2002). Committee on infectious diseases. Head lice. Pediatrics.

[133] Ali A, Ahmad S, de Albuquerque PMM, Kamil A, Alshammari FA, Alouffi A (2021). Prediction of novel drug targets and vaccine candidates against human lice (Insecta), acari (Arachnida), and their associated pathogens. Vaccines (Basel).

[134] Diamantis SA, Morrell DS, Burkhart CN (2009). Treatment of head lice. Dermatol Therapy.

[135] Yamaguchi S, Yasumura R, Okamoto Y, Okubo Y, Miyagi T, Kawada H (2021). Efficacy and safety of a dimethicone lotion in patients with pyrethroid-resistant head lice in an epidemic area, Okinawa. Jpn J Dermatol.

[136] Martínez de Murguía Fernández L, Puig Algora G, BajonaRoig M, Bacchini G (2021). Effectiveness and tolerability of a squalane and dimethicone-based treatment for head lice. Parasitol Res..

[137] Ihde ES, Boscamp JR, Loh JM, Rosen L (2015). Safety and efficacy of a 100% dimethicone pediculocide in school-age children. BMC Pediatr.

[138] Lamassiaude N, Toubate B, Neveu C, Charnet P, Dupuy C, Debierre-Grockiego F (2021). The molecular targets of ivermectin and lotilaner in the human louse *Pediculus*
*humanus*
*humanus*: new prospects for the treatment of pediculosis. PLoS Pathog.

[139] Lebwohl M, Clark L, Levitt J (2007). Therapy for head lice based on life cycle, resistance, and safety considerations. Pediatrics.

[140] Idriss S, Levitt J (2009). Malathion for head lice and scabies: treatment and safety considerations. J Drugs Dermatol.

[141] Burkhart CG (2004). Relationship of treatment-resistant head lice to the safety and efficacy of pediculicides. Mayo Clin Proc.

[142] Villegas SC, Breitzka RL (2012). Head lice and the use of spinosad. Clin Ther.

[143] Deeks LS, Naunton M, Currie MJ, Bowden FJ (2013). Topical ivermectin 0.5% lotion for treatment of head lice. Ann Pharmacother..

[144] Feldmeier H (2014). Treatment of pediculosis capitis: a critical appraisal of the current literature. Am J Clin Dermatol.

[145] Leulmi H, Diatta G, Sokhna C, Rolain JM, Raoult D (2016). Assessment of oral ivermectin versus shampoo in the treatment of pediculosis (head lice infestation) in rural areas of Sine-Saloum, Senegal. Int J Antimicrob Agents.

[146] Chosidow O, Giraudeau B, Cottrell J, Izri A, Hofmann R, Mann SG (2010). Oral ivermectin versus malathion lotion for difficult-to-treat head lice (Erratum in: N Engl J Med. 2010;362:1647). N Engl J Med..

