# Supplementary material for: Human pediculosis, a global public health problem
Source: Infect Dis Poverty. 2022 May 26;11:58. doi: 10.1186/s40249-022-00986-w (PMC9134731; doi:10.1186/s40249-022-00986-w)
Supplement: Supplementary file 1 — Additional file 1: Table S1. Summary of the main genetic studies reported on human head and body lice. Table S2. Species of head lice and body lice included in mt cytb gene phylogenetic analysis. [file 40249_2022_986_MOESM1_ESM.docx]

**Table S1** Summary of the main genetic studies on human head and body lice

| DNA type | Gene | Fragment length | Reference |
| --- | --- | --- | --- |
| Mitochondrial DNA | Cytochrome *c* oxidase subunit 1 (*Cox*1) | 524 bp | [1] |
|  |  | 610 bp | [2] |
|  |  | 524 bp | [3] |
|  |  | 854 bp | [4] |
|  |  | 383 bp | [5] |
|  |  | 827 bp | [6] |
|  |  | 379 bp | [7] |
|  |  | 658 bp | [8] |
|  |  | 599−603 bp | [9] |
|  |  | 283−286 bp | [10] |
|  |  | 283 bp | [11] |
|  |  | 828 bp | [12] |
|  |  | 599−603 bp | [13] |
|  | Cytochrome b (*Cyt*b) | 440 bp | [2] |
|  |  | 671 bp | [4] |
|  |  | 356 bp | [5] |
|  |  | 316 bp | [14] |
|  |  | 316 bp | [15] |
|  |  | 270 bp | [16] |
|  |  | 347 bp | [17] |
|  |  | 294 bp | [8] |
|  |  | 360 bp | [18] |
|  |  | 347 bp | [19] |
|  |  | 347 bp | [20] |
|  |  | 88 bp | [21] |
|  |  | 272 bp | [10] |
|  |  | 347 bp | [22] |
|  |  | 347 bp | [23] |
|  |  | 270 bp | [24] |
|  |  | 347 bp | [11] |
|  |  | 347 bp | [25] |
|  |  | 347 bp | [26] |
|  |  | 348 bp | [12] |
|  |  | 347 bp | [27] |
|  |  | 347 bp | [28] |
|  |  | 347 bp | [29] |
|  |  | 347 bp | [30] |
|  |  | 347 bp | [31] |
|  | NADH dehydrogenase 2 (*Nad*2) | 839 bp | [10] |
|  | NADH dehydrogenase 4 (*Nad*4) | 579 bp | [2] |
|  | 12S ribosomal RNA | 100 bp | [21] |
|  |  | 596 bp | [12] |
|  | 16S ribosomal RNA | 667−668 bp | [10] |
| Nuclear DNA | Elongation factor 1α (*EF-1α*) | 485 bp | [2] |
|  |  | 348 bp | [3] |
|  | RNA polymerase II (*RPII*) | 601 bp | [2] |
|  |  | 279 bp | [10] |
|  | 18S rRNA gene, small ribosomal subunit rRNA | 1,474–1,493 bp | [3] |
|  |  | 1,195 bp | [32] |
|  |  | 467−475 bp | [10] |
|  |  | 1,474–1,493 bp | [24] |
|  | Glycerol-3-phosphate dehydrogenase (*GPD*) | 688−706 bp | [10] |
|  | Microsatellites | 130–180 bp | [33] |
|  |  | 103−392 bp  107−347 bp  119−220 bp  117−236 bp  223−248 bp  144−247 bp  118−196 bp  173−248 bp  132−300 bp | [34] |
|  | Intergenic spacers | 133–155 bp  323–328 bp  165–185 bp  156–189 bp | [14] |
|  |  | 215−229 bp  212−216 bp  324 bp | [35] |
|  |  | 217 bp  437 bp  488 bp  492 bp | [18] |
|  |  | 216–223 bp  323−324 bp | [10] |

**Table S2** Species of human head lice and body lice included in the mt *cyt*b gene phylogenetic analysis

| Clade number | GenBank accession | Ecotype | Country/Region | Reference |
| --- | --- | --- | --- | --- |
| A1 | MF672002 | *Pediculus humanus capitis* | France | [11] |
| A2 | KX444540 | *Pediculus humanus capitis* | Republic of the Congo | [20] |
| A3 | KX444542 | *Pediculus humanus capitis* | Republic of the Congo | [20] |
| A4 | KX444539 | *Pediculus humanus capitis* | Republic of the Congo | [20] |
| A5 | KM579568 | *Pediculus humanus capitis* | Brazil | [10] |
| A6 | MH230926 | *Pediculus humanus capitis* | Democratic Republic of the Congo | [28] |
| A7 | MF672003 | *Pediculus humanus capitis* | France | [11] |
| A8 | KM579544 | *Pediculus humanus humanus* | Peru | [10] |
| A9 | KM579539 | *Pediculus humanus humanus* | Russia | [10] |
| A10 | KM579550 | *Pediculus humanus humanus* | Asia | [10] |
| B1 | MF672004 | *Pediculus humanus capitis* | France | [11] |
| B2 | KM579556 | *Pediculus humanus capitis* | Honduras | [10] |
| B3 | AY696013 | *Pediculus humanus capitis* | USA | [4] |
| B4 | AY316764 | *Pediculus humanus capitis* | Germany | [2] |
| B5 | AY316778 | *Pediculus humanus capitis* | UK | [2] |
| B6 | AY69601 | *Pediculus humanus capitis* | Honduras | [4] |
| B7 | AY696009 | *Pediculus humanus capitis* | USA | [4] |
| B8 | AY696015 | *Pediculus humanus capitis* | Honduras | [4] |
| B9 | AY696010 | *Pediculus humanus capitis* | USA | [4] |
| B10 | AY696014 | *Pediculus humanus capitis* | USA | [4] |
| C1 | KX444552 | *Pediculus humanus capitis* | Republic of the Congo | [20] |
| C2 | KM579563 | *Pediculus humanus capitis* | Ethiopia | [10] |
| C3 | KX444547 | *Pediculus humanus capitis* | Republic of the Congo | [20] |
| C4 | KM579562 | *Pediculus humanus capitis* | Nepal | [10] |
| C5 | KM579561 | *Pediculus humanus capitis* | Ethiopia | [10] |
| C6 | AY316767 | *Pediculus humanus capitis* | Ethiopia | [2] |
| C7 | AY316765 | *Pediculus humanus capitis* | Ethiopia | [2] |
| C8 | AY316766 | *Pediculus humanus capitis* | Ethiopia | [2] |
| C9 | JF694406 | *Pediculus humanus capitis* | Ethiopia | [16] |
| C10 | KM579565 | *Pediculus humanus capitis* | Ethiopia | [10] |
| D1 | MH230923 | *Pediculus humanus capitis* | Democratic Republic of the Congo | [28] |
| D2 | KX249773 | *Pediculus humanus capitis* | Democratic Republic of the Congo | [21] |
| D3 | KX249768 | *Pediculus humanus capitis* | Democratic Republic of the Congo | [21] |
| D4 | MH230924 | *Pediculus humanus capitis* | Democratic Republic of the Congo | [28] |
| D5 | MH230922 | *Pediculus humanus capitis* | Democratic Republic of the Congo | [28] |
| D6 | KX249774 | *Pediculus humanus capitis* | Ethiopia | [21] |
| D7 | KX249771 | *Pediculus humanus capitis* | Democratic Republic of the Congo | [21] |
| D8 | KX444544 | *Pediculus humanus capitis* | Republic of the Congo | [20] |
| D9 | MH117951 | *Pediculus humanus humanus* | Democratic Republic of the Congo | Unpublished |
| D10 | MH117947 | *Pediculus humanus humanus* | Democratic Republic of the Congo | Unpublished |
| E1 | MF672006 | *Pediculus humanus capitis* | France | [11] |
| E2 | MG759556 | *Pediculus humanus capitis* | France | [11] |
| E3 | MH429050 | *Pediculus humanus capitis* | Nigeria | [12] |
| E4 | MT981019 | *Pediculus humanus capitis* | Guinea | [31] |
| E5 | KY937989 | *Pediculus humanus capitis* | Mali | [23] |
| E6 | MT981014 | *Pediculus humanus capitis* | Guinea | [31] |
| E7 | KM579560 | *Pediculus humanus capitis* | Senegal | [10] |
| E8 | MH230921 | *Pediculus humanus capitis* | Democratic Republic of the Congo | [28] |
| E9 | KY937990 | *Pediculus humanus capitis* | Mali | [23] |
| E10 | MT981015 | *Pediculus humanus capitis* | Guinea | [31] |
| F1 | MH429040 | *Pediculus humanus capitis* | Amazonia | [12] |
| F2 | MH429041 | *Pediculus humanus capitis* | Amazonia | [12] |
| F3 | MH429042 | *Pediculus humanus capitis* | Amazonia | [12] |
| F4 | MH429043 | *Pediculus humanus capitis* | Amazonia | [12] |
| F5 | MH429044 | *Pediculus humanus capitis* | Amazonia | [12] |
| F6 | MH429045 | *Pediculus humanus capitis* | Amazonia | [12] |
| F7 | MH429046 | *Pediculus humanus capitis* | Amazonia | [12] |
| F8 | MH429039 | *Pediculus humanus capitis* | Amazonia | [12] |

**References**

1. Leo NP, Campbell NJ, Yang X, Mumcuoglu K, Barker SC. Evidence from mitochondrial DNA that head lice and body lice of humans (Phthiraptera: Pediculidae) are conspecific. J Med Entomol. 2002;39:662-6.
2. Kittler R, Kayser M, Stoneking M. Molecular evolution of *Pediculus humanus* and the origin of clothing. Curr Biol. 2003;13:1414-7.
3. Yong Z, Fournier PE, Rydkina E, Raoult D. The geographical segregation of human lice preceded that of *Pediculus humanus capitis* and *Pediculus humanus humanus*. C R Biol. 2003;326:565-74.
4. Reed DL, Smith VS, Hammond SL, Rogers AR, Clayton DH. Genetic analysis of lice supports direct contact between modern and archaic humans. PLoS Biol. 2004;2:e340.
5. Raoult D, Reed DL, Dittmar K, Kirchman JJ, Rolain JM, Guillen S, et al. Molecular identification of lice from pre-Columbian mummies. J Infect Dis. 2008;197:535-43.
6. Light JE, Allen JM, Long LM, Carter TE, Barrow L, Suren G, et al. Geographic distributions and origins of human head lice (*Pediculus humanus capitis*) based on mitochondrial data. J Parasitol. 2008;94:1275–81.
7. Ascunce MS, Fane J, Kassu G, Toloza AC, Picollo MI, González-Oliver A, et al. Mitochondrial diversity in human head louse populations across the Americas. Am J Phys Anthropol. 2013;152:118-29.
8. Ashfaq M, Prosser S, Nasir S, Masood M, Ratnasingham S, Hebert PD. High diversity and rapid diversifification in the head louse, *Pediculus humanus* (Pediculidae: Phthiraptera). Sci Rep. 2015;5:14188.
9. Sunantaraporn S, Sanprasert V, Pengsakul T, Phumee A, Boonserm R, Tawatsin A, et al. Molecular survey of the head louse *Pediculus humanus capitis* in Thailand and its potential role for transmitting *Acinetobacter* spp. Parasit Vectors. 2015;8:127.
10. Drali R, Abi-Rached L, Boutellis A, Djossou F, Barker SC, Raoult D. Host switching of human lice to new world monkeys in South America. Infect Genet Evol. 2016;39:225-31.
11. Candy K, Amanzougaghene N, Izri A, Brun S, Durand R, Louni M, et al. Molecular survey of head and body lice, *Pediculus humanus*, in France. Vector Borne Zoonotic Dis. 2018;18:243–51.
12. Amanzougaghene N, Fenollar F, Davoust B, Djossou F, Ashfaq M, Bitam I, et al. Mitochondrial diversity and phylogeographic analysis of *Pediculus humanus* reveals a new Amazonian clade "F". Infect Genet Evol. 2019;70:1-8.
13. Mokhtar AS, Ling Lau Y, Wilson JJ, Abdul-Aziz NM. Genetic diversity of *Pediculus humanus capitis* (Phthiraptera: Pediculidae) in Peninsular Malaysia and molecular detection of its potential associated pathogens. J Med Entomol. 2020;57:915-26.
14. Li W, Ortiz G, Fournier PE, Gimenez G, Reed DL, Pittendrigh B, et al. Genotyping of human lice suggests multiple emergencies of body lice from local head louse populations. PLoS Negl Trop Dis. 2010;4:e641.
15. Boutellis A, Veracx A, Angelakis E, Diatta G, Mediannikov O, Trape JF, et al. *Bartonella quintana* in head lice from Sénégal. Vector Borne Zoonotic Dis. 2012;12:564-7.
16. Boutellis A, Drali R, Rivera MA, Mumcuoglu KY, Raoult D. Evidence of sympatry of clade a and clade B head lice in a pre-Columbian Chilean mummy from Camarones. PLoS One. 2013;8:e76818.
17. Sangaré AK, Boutellis A, Drali R, Socolovschi C, Barker SC, Diatta G, et al. Detection of *Bartonella quintana* in African body and head lice. Am J Trop Med Hyg. 2014;91:294-301.
18. Boutellis A, Bitam I, Fekir K, Mana N, Raoult D. Evidence that clade A and clade B head lice live in sympatry and recombine in Algeria. Med Vet Entomol. 2015;29:94-8.
19. Drali R, Shako JC, Davoust B, Diatta G, Raoult D. A new clade of African body and head lice infected by *Bartonella quintana* and *Yersinia pestis*-Democratic Republic of the Congo. Am J Trop Med Hyg. 2015;93:990–3.
20. Amanzougaghene N, Akiana J, Mongo Ndombe G, Davoust B, Nsana NS, Parra HJ, et al. Head lice of pygmies reveal the presence of relapsing fever borreliae in the Republic of Congo. PLoS Negl Trop Dis. 2016;10:e0005142.
21. Amanzougaghene N, Mumcuoglu KY, Fenollar F, Alfi S, Yesilyurt G, Raoult D, et al. High ancient genetic diversity of human lice, *Pediculus humanus*, from Israel reveals new insights into the origin of clade B lice. PLoS One. 2016;11:e0164659.
22. Al-Shahrani SA, Alajmi RA, Ayaad TH, Al-Shahrani MA, Shaurub EH. Genetic diversity of the human head lice, *Pediculus humanus capitis*, among primary school girls in Saudi Arabia, with reference to their prevalence. Parasitol Res. 2017;116:2637-43.
23. Amanzougaghene N, Fenollar F, Sangaré AK, Sissoko MS, Doumbo OK, Raoult D, et al. Detection of bacterial pathogens including potential new species in human head lice from Mali. PLoS One. 2017;12:e0184621.
24. Mana N, Louni M, Parola P, Bitam I. Human head lice and pubic lice reveal the presence of several *Acinetobacter* species in Algiers, Algeria. Comp Immunol Microbiol Infect Dis. 2017;53:33-9.
25. Louni M, Mana N, Bitam I, Dahmani M, Parola P, Fenollar F, et al. Body lice of homeless people reveal the presence of several emerging bacterial pathogens in northern Algeria. PLoS Negl Trop Dis. 2018;12:e0006397.
26. Louni M, Amanzougaghene N, Mana N, Fenollar F, Raoult D, Bitam I, et al. Detection of bacterial pathogens in clade E head lice collected from Niger's refugees in Algeria. Parasit Vectors. 2018;11:348.
27. De Liberato C, Magliano A, Romiti F, Menegon M, Mancini F, Ciervo A, et al. Report of the human body louse (*Pediculus humanus*) from clothes sold in a market in central Italy. Parasit Vectors. 2019;12:201.
28. Boumbanda Koyo CS, Amanzougaghene N, Davoust B, Tshilolo L, Lekana-Douki JB, Raoult D, et al. Genetic diversity of human head lice and molecular detection of associated bacterial pathogens in Democratic Republic of Congo. Parasit Vectors. 2019;12:290.
29. Amanzougaghene N, Mediannikov O, Ly TDA, Gautret P, Davoust B, Fenollar F, et al. Molecular investigation and genetic diversity of *Pediculus* and *Pthirus* lice in France. Parasit Vectors. 2020;13:177.
30. Boumbanda-Koyo CS, Mediannikov O, Amanzougaghene N, Oyegue-Liabagui SL, Imboumi-Limoukou RK, Raoult D, et al. Molecular identification of head lice collected in Franceville (Gabon) and their associated bacteria. Parasit Vectors. 2020;13:410.
31. Hammoud A, Louni M, Baldé MC, Beavogui AH, Gautret P, Raoult D, et al. Molecular characterization and genetic diversity of haplogroup E human lice in Guinea, West Africa. Microorganisms. 2021;9:257.
32. Leo NP, Barker SC. Unravelling the evolution of the head lice and body lice of humans. Parasitol Res. 2005;98:44-7.
33. Leo NP, Hughes JM, Yang X, Poudel SK, Brogdon WG, Barker SC. The head and body lice of humans are genetically distinct (Insecta: Phthiraptera, Pediculidae): evidence from double infestations. Heredity (Edinb). 2005;95:34-40.
34. Ascunce MS, Toups MA, Kassu G, Fane J, Scholl K, Reed DL. Nuclear genetic diversity in human lice (*Pediculus humanus*) reveals continental differences and high inbreeding among worldwide populations. PLoS One. 2013;8:e57619.
35. Veracx A, Rivet R, McCoy KD, Brouqui P, Raoult D. Evidence that head and body lice on homeless persons have the same genotype. PLoS One. 2012;7:e45903.
